# Perturbation of the host cell Ca^2+^ homeostasis and ER-mitochondria contact sites by the SARS-CoV-2 structural proteins E and M

**DOI:** 10.1038/s41419-023-05817-w

**Published:** 2023-04-29

**Authors:** Elena Poggio, Francesca Vallese, Andreas J. W. Hartel, Travis J. Morgenstern, Scott A. Kanner, Oliver Rauh, Flavia Giamogante, Lucia Barazzuol, Kenneth L. Shepard, Henry M. Colecraft, Oliver Biggs Clarke, Marisa Brini, Tito Calì

**Affiliations:** 1 Department of Biology, University of Padova, Padova, Italy; 2 Department of Anesthesiology, Columbia University Irving Medical Center, New York, NY USA; 3 Department of Physiology and Cellular Biophysics, Columbia University, New York, NY USA; 4 Department of Electrical Engineering, Columbia University, New York, NY USA; 5 Department of Molecular Pharmacology and Therapeutics, Columbia University Irving Medical Center, New York, NY USA; 6 Doctoral Program in Neurobiology and Behavior, Columbia University Vagelos College of Physicians and Surgeons, New York, NY USA; 7 Membrane Biophysics, Department of Biology, Technical University of Darmstadt, Darmstadt, Germany; 8 Department of Biomedical Sciences, University of Padova, Padova, Italy; 9 Study Center for Neurodegeneration (CESNE), University of Padova, Padova, Italy; 10 Padova Neuroscience Center (PNC), University of Padova, Padova, Italy

**Keywords:** Ion channels, Infectious diseases

## Abstract

Coronavirus disease (COVID-19) is a contagious respiratory disease caused by the SARS-CoV-2 virus. The clinical phenotypes are variable, ranging from spontaneous recovery to serious illness and death. On March 2020, a global COVID-19 pandemic was declared by the World Health Organization (WHO). As of February 2023, almost 670 million cases and 6,8 million deaths have been confirmed worldwide. Coronaviruses, including SARS-CoV-2, contain a single-stranded RNA genome enclosed in a viral capsid consisting of four structural proteins: the nucleocapsid (N) protein, in the ribonucleoprotein core, the spike (S) protein, the envelope (E) protein, and the membrane (M) protein, embedded in the surface envelope. In particular, the E protein is a poorly characterized viroporin with high identity amongst all the β-coronaviruses (SARS-CoV-2, SARS-CoV, MERS-CoV, HCoV-OC43) and a low mutation rate. Here, we focused our attention on the study of SARS-CoV-2 E and M proteins, and we found a general perturbation of the host cell calcium (Ca^2+^) homeostasis and a selective rearrangement of the interorganelle contact sites. In vitro and in vivo biochemical analyses revealed that the binding of specific nanobodies to soluble regions of SARS-CoV-2 E protein reversed the observed phenotypes, suggesting that the E protein might be an important therapeutic candidate not only for vaccine development, but also for the clinical management of COVID designing drug regimens that, so far, are very limited.

## Introduction

β-coronaviruses (such as SARS-CoV, MERS-CoV, HCoV-OC43 and SARS-CoV-2) are one of the four members (α-,β-,γ- and δ-) of the *Coronaviridae* family characterized by zoonotic transmission [1] and spread among humans through close contact [2, 3]. Infection with SARS-CoV-2 causes coronavirus disease (COVID-19), a contagious respiratory disease whose symptoms include fever, headache, cough, anosmia and ageusia, respiratory distress, systemic inflammation, cardiac injury, and multi-organ dysfunction in high-risk individuals [4]. The ~30 kilobases long positive-sense single-stranded RNA genome of SARS-CoV-2 encodes 4 structural proteins, 16 non-structural proteins (nsp1–16) and 9 accessory proteins sharing about 80% sequence identity with SARS-CoV and MERS-CoV and >90% sequence identity with viral essential enzymes and structural proteins [5] indicative of a common pathogenic mechanism [6]. Structurally, SARS-CoV-2 contains four major proteins: the spike (S), envelope (E), and membrane (M) proteins are embedded in the viral surface, while the nucleocapsid (N) protein is in the ribonucleoprotein core. They are responsible for i) recognition of the host cellular receptor, ii) virus assembly, budding and infectivity, iii) shaping the virion envelope and iv) binding to the viral RNA and the formation of viral particles (for N, E and M), respectively.

In the viral envelope, the S protein is an homotrimer consisting of a distal and a proximal subunit, which is cleaved by the target cell proteases into the S1 and S2 subunits to allow viral entry [7, 8]. The receptor-binding domain (RBD) on the S1 subunit determines receptor recognition, whereas the S2 subunit containing the fusion peptide (FP), a connecting region (CR) and repeat regions (HR1 and HR2) [9], is responsible for the virus entry via membrane fusion [10]. The E and M structural proteins are also embedded in the viral surface, and play a role in shaping the viral envelope and in the assembly, invasion, replication, and release of the virus [11]. Nevertheless, their specific role, as well as their action on the host cell machinery, is still almost entirely unexplored. The M protein interacts with itself and with the S protein through one of its three transmembrane domains. In addition, its C-terminal region faces the inner side of the viral particle and interacts with the N and E proteins to promote the formation of the virus-like particles core, membrane bending, virus assembly and germination [12–15]. SARS- CoV-2 E protein is a small 75 amino acids integral membrane protein with one transmembrane domain (residues 17–37), an intermediate helical domain, and hydrophilic N- and C-terminal domains [16–18]. However, the membrane topology of E within the host cell is still a matter of debate, as its orientation appears dependent on the expression level and oligomerization state [19]. Among the structural proteins, the E protein is the most enigmatic and the least characterized. Functionally it may act as a virulence factor [20, 21], and its interaction with the M protein is required to promote the release of viral particles [22], as well as to direct the correct trafficking of the S protein towards the secretory pathway [23]. Most of the protein is located at intracellular transport sites involved in CoV assembly and budding, e.g., the ER, Golgi and ERGIC [24]. Studies have revealed that the central transmembrane region of the E protein oligomerizes to form pentameric ion channels whose activity is required for viral pathogenicity [25–29] and, therefore probably also crucial in regulating the ion balance of host cells. Accordingly, the E protein is a viroporin, normally used by RNA viruses to subvert the host cell ion homeostasis to facilitate viral infection [30, 31], forming a cation-selective voltage-dependent channel across the ERGIC membrane [32, 33]. The cation permeability of the E protein can change based on ions concentration (K^+^, Na^+^, Ca^2+^ and Mg^2+^) [32, 34], composition of the cell membrane [33, 35] and pH [30]. SARS-CoV-2 has also been shown to perturb host cell Ca^2+^ homeostasis highlighting the critical role of Ca^2+^ for infection and pathogenesis [34]. Therefore, ion leakage via the E protein might play an important role in the development of respiratory inflammation via inflammasome activation [29, 36]. Importantly, the sequence identity of M or E proteins among β-coronaviruses is much higher than the one for the S protein and RBD, suggesting a strong potential in cross-protection if targeted by vaccines. Although the global efforts aimed at the SARS-CoV-2 proteins for vaccine development have helped reducing the burden of the disease [37], the S protein is the main protein used as a target while other structural proteins remain neglected and have never been explored as targets for vaccines or drugs [38–40]. Nevertheless, the molecular pathways by which SARS-CoV-2 hijacks the host cell molecular machinery for its entry, replication, and egress are still poorly characterized. It is therefore fundamental to identify the mechanisms that could be therapeutically targeted to halt SARS-CoV-2 infection and COVID-19 pathogenesis. Here we explored the role of SARS-CoV-2 M and E proteins on the overall cellular Ca^2+^ signaling and found that different mechanisms of action are in place to selectively subvert ER and mitochondria Ca^2+^ handling, respectively. Additionally, to overcome the low immunogenicity of the SARS-CoV-2 E protein (possibly due to its small size and absence of ectodomains for immune cell recognition) [38, 39], specific nanobodies against the SARS-CoV-2 E protein were generated, isolated, and tested. In vitro electrophysiological recordings and in vivo biochemical analysis revealed the efficacy of the nanobodies in targeting the E protein’s function highlighting the potential role of the SARS-CoV-2 E protein viroporin as vaccine target and pointing to ER and mitochondrial Ca^2+^ handling as potential therapeutic targets to halt SARS-CoV-2 infection and COVID-19 pathogenesis.

## Results

### SARS-CoV-2 E and M proteins expression and subcellular localization in mammalian HeLa cells

**Fig. 1:**
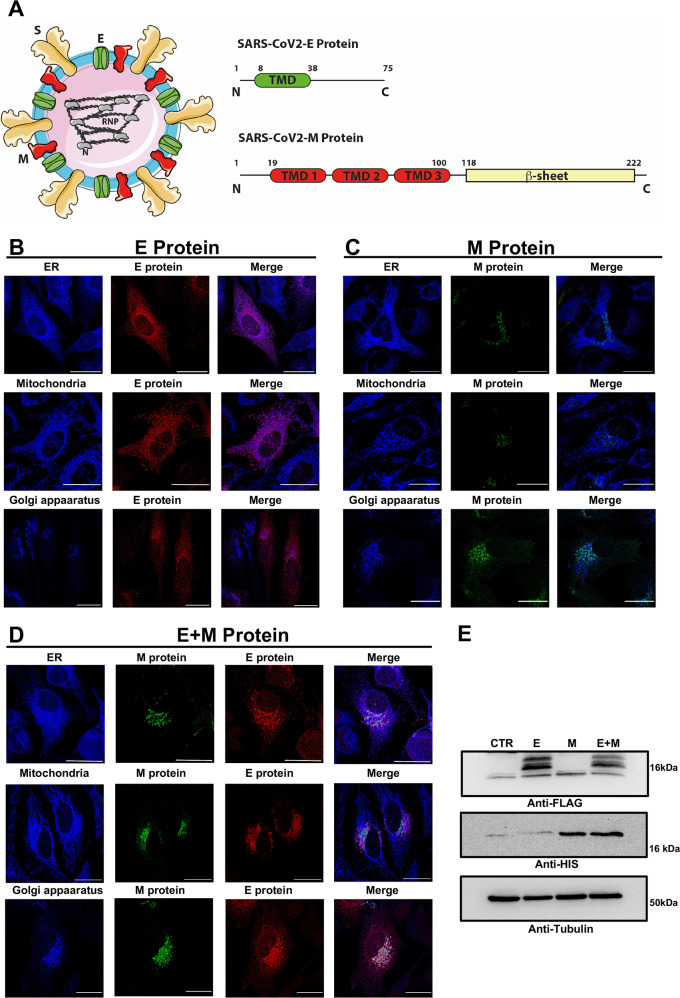
SARS-CoV-2 structure and subcellular localization of the E and M proteins in host cells. **A** Cartoon showing the arrangement of the SARS-CoV-2 structural proteins and the domain organization of the E and M structural proteins. **B**–**D** The SARS-CoV-2 E and M proteins were transfected, either alone (**B** and **C**, respectively) or in co-transfection (**D**) in HeLa cells. After fixation, immunofluorescence staining was performed to detect the presence of the viral proteins and their colocalization with subcellular compartments. In particular, we used a rabbit polyclonal antibody against the FLAG to detect the E protein and a mouse monoclonal antibody against the His tag to reveal the M protein. To stain the subcellular compartments, we used a rabbit polyclonal antibody anti-KDEL for the ER, a rabbit polyclonal antibody anti-TOM20 for mitochondria, and a rabbit polyclonal antibody to TGN46, as a Golgi marker. Images were acquired with a ZEISS LSM700 confocal microscope with an EC “Plan-Neofluar” 63x/0.50 M27 objective upon illumination with laser at the wavelength of 405 nm, 488 nm, and 594 nm. Scalebar = 25 µM. **E** A representative western blotting analysis of HeLa cells expressing the SARS-CoV-2 E and M proteins, either alone or in co-transfection. To detect the E and M protein bands, we used a rabbit polyclonal antibody anti-FLAG tag and a mouse monoclonal antibody anti-His tag, respectively. To verify the proper protein loading, we used an anti-β tubulin antibody.

Together with the S protein, the E and M proteins are the only structural SARS-CoV-2 proteins of the viral envelope (Fig. 1A left). The E protein is a small, 75 amino acid-long, protein with a single transmembrane domain and the C-terminal domain facing the inside of the viral membrane. The NMR structural models of E protein in detergent micelle and lipid bilayer provide evidence that the protein assembles into higher- order oligomers to form a pentameric, ion selective viroporin [26, 30]. The M protein is larger, 220 amino acid-long, and consists of three transmembrane helices and a β-sheet region at the C-terminus that faces the inside of the viral particle. The M protein mediates the concerted recruitment of the N protein and RNA components through the positively charged intravirion domain (Fig. 1A). The cryo-EM structure of the SARS-CoV2 M protein has been recently solved in two different conformations. M protein forms a dimer [41] but can also further assemble into higher-order oligomers [42]. Although their specific role is still largely unclear, M/E, M/N or M/N/E interactions are important for efficient virion assembly [43]. First, we have expressed the E and M proteins in mammalian HeLa cells, either alone or in combination (E + M), to explore their subcellular localization by immunofluorescence analyses (Fig. 1B–D). The overexpressed Flag-tagged E protein is present throughout the cell in secretory/vesicular compartments, possibly representing ER/ERGIC compartments, as already proposed [10, 43] (Fig. 1B), while the overexpressed His-tagged M protein is more specifically localized (Fig. 1C). Double immunofluorescence analysis with endogenous ER (KDEL), mitochondrial (TOM20) and Golgi apparatus (TGN46) markers revealed partial colocalization with ER and Golgi markers for the E protein and almost complete colocalization with Golgi marker for the M protein (Fig. 1E). Interestingly, when co-expressed the E and M proteins co-localized at the Golgi apparatus and the E protein was less diffusely distributed, suggesting that the E protein subcellular localization might be influenced by the presence of the M protein (Fig. 1D–E). Western Blot analysis confirmed their expression and migration pattern (Fig. 1F).

### Subversion of host HeLa cells Ca^2+^-signaling pathways by SARS-CoV-2 E and M proteins overexpression

**Fig. 2:**
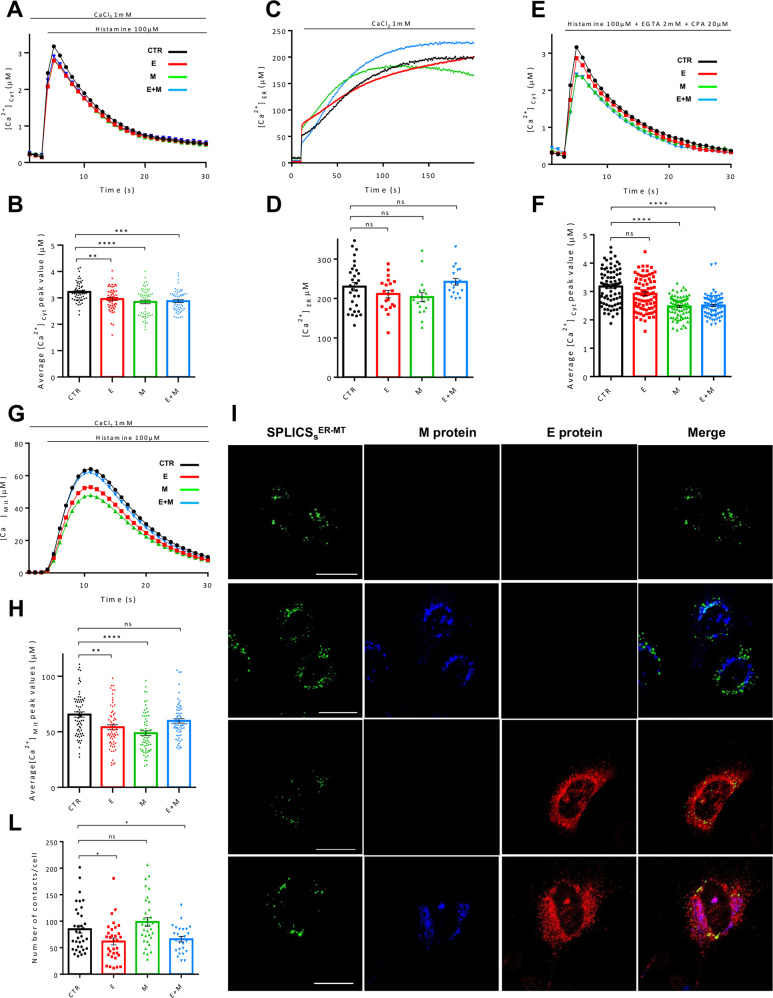
SARS-CoV-2 E and M proteins affect the calcium handlings and inter-organelle contact sites of the host cells. **A** HeLa cells were transfected with empty vector/the E and M proteins, either alone or in co-transfection, and the cytosolic-localized aequorin. Then, cells were stimulated with 100 μM histamine to elicit a cytosolic Ca^2+^ transient. From each calcium trace (average of at least 60 independent measurements obtained from at least three independent transfections, mean ± SEM), the peak value was calculated and plotted in panel **B** (mean ± SEM). One-way ANOVA = ****p < 0.001. Sidak’s multiple comparisons test was applied for multiple comparisons (**p < 0.01; ***p < 0.001; *p* < 0.0001 versus CTR). **C** To evaluate the ER Ca^2+^ basal levels, HeLa cells were transfected with a low affinity ER-targeted aequorin and the viral proteins/empty vector. First, the ER Ca^2+^ content was drastically reduced by incubating cells with 5 μM ionomycin, and 600 μM EGTA; then, Ca^2+^ was re-introduced (3 mM CaCl2) to monitor the ER Ca^2+^ uptake. From each calcium trace (average of at least 15 independent measurements obtained from three independent transfections, mean ± SEM), the plateau value was calculated and plotted in panel **D** (mean ± SEM). Kruskal-Wallis test = *p < 0.05. Dunn’s multiple comparisons test was applied for multiple comparisons. **E** To measure the ER Ca^2+^ release, HeLa cells, transfected with the cytosolic aequorin and the viral proteins/empty vector, were incubated in a KRB solution with a low Ca^2+^ concentration (0.5 mM) and, then, stimulated with a KRB solution consisting of 100 μM histamine to induce the ER Ca^2+^ release, 40 µM CPA to inhibit the SERCA pump, and EGTA 4 mM to chelate the external Ca^2+^. From each calcium trace (average of at least 60 independent measurements obtained from three independent transfections, mean ± SEM), the peak value was calculated and plotted in panel **F** (mean ± SEM). Kruskal-Wallis test = *p < 0.05. Dunn’s multiple comparisons test was applied for multiple comparisons (*p* < 0.001; ****p < 0.0001 versus CTR). **G** HeLa cells were transfected with the E and M proteins, either alone or in co-transfection, and the mitochondrial-targeted mutated aequorin. Then, cells were stimulated with 100 μM histamine to induce the mitochondrial Ca^2+^ uptake. From each calcium trace (average of at least 60 independent measurements obtained from at least three independent transfections, mean ± SEM), the peak was calculated and plotted in panel **H** (mean ± SEM). One-way ANOVA = ****p < 0.0001. Tukey’s multiple comparisons test was applied for multiple comparisons (**p < 0.01; ****p < 0.0001 versus CTR). **I** Representative images of HeLa cells expressing the SPLICSS-P2A ER–MT with or without the E and M proteins (detected by a rabbit polyclonal antibody anti-FLAG tag and a mouse monoclonal antibody anti-His tag, respectively). Images were acquired with a ZEISS LSM700 confocal microscope with an EC “Plan-Neofluar” 63x/0.50 M27 objective upon illumination with laser at the wavelength of 405 nm, 488 nm, and 594 nm. **L** Quantification of SPLICS dots was performed with the ImageJ software, after the 3D rendering reconstitution of the complete Z-stacks. The number of contacts per cell is expressed by the mean ± SEM, and the data were obtained from three independent transfections. One-way ANOVA = ***p ≤ 0.001. Fisher’s LSD multiple comparisons test was applied for multiple comparisons (*p < 0.05 versus CTR).

To explore the effect of the SARS-CoV-2 E and M proteins on the Ca^2+^ signaling pathways, HeLa cells were transfected with the Flag-tagged E and the His-tagged M proteins, either alone (E and M) or in combination (E + M), along with a vector encoding the cytosolic photoprotein aequorin (cytAEQ). Ca^2+^ analyses were performed stimulating cells with the InsP3-linked agonist histamine (100 μM) to induce Ca^2+^ release from the ER and recording the resulting aequorin luminescence signals with a PerkinElmer EnVision plate reader. As shown by the Ca^2+^ transients in Fig. 2A and the Ca^2+^ peaks quantified in Fig. 2B, cells overexpressing the SARS-CoV-2 E, M and E + M proteins, cleared the histamine-induced cytosolic Ca^2+^ transient slightly more efficiently than control cells (CTR) (peak values μM ± SEM: 3.225 ± 0.049, *n* = 57 for CTR; 2.956 ± 0.058, *n* = 56 for SARS-CoV-2 E; 2.848 ± 0.068, *n* = 59 for SARS-CoV-2 M; 2.878 ± 0.053, *n* = 57 for SARS-CoV-2 E + M), while no statistically significant differences were found comparing E, M and E + M to each other. Although these results could suggest a possible positive modulation of some Ca^2+^-exporting systems or negative regulation of the Ca^2+^-import machinery by the SARS-CoV-2 E and M proteins, it is unlikely that the decrease in cytosolic Ca^2+^ peak values compared to CTR cells could strongly impinge on the general host cells Ca^2+^ signaling. Therefore, we have assessed whether the expression of the SARS-CoV-2 E, M and E + M proteins impacts on the ER Ca^2+^ levels. Thus, we expressed the ER targeted low affinity aequorin (erAEQ) in HeLa cells, either alone or together with the E, the M, and the E + M proteins. As shown in the representative traces in Fig. 2C and in the Ca^2+^ levels quantified in Fig. 2D, the maximum [Ca^2+^]_ER_ reached in CTR cells and in cells expressing the SARS-CoV-2 E, M and E + M proteins, was statistically unaltered (plateau values μM ± SEM: 230.1 ± 10.77, *n* = 30 for CTR; 211.2 ± 9.5, *n* = 20 for SARS-CoV-2 E; 203.4 ± 11.22, *n* = 18 for SARS-CoV-2 M; 242.1 ± 8.43, *n* = 18 for SARS-CoV-2 E + M), suggesting that neither the ability of the cell to refill ER Ca^2+^ content nor the ER Ca^2+^ basal levels were affected by overexpression of the E, M or E + M proteins. The cytosolic Ca^2+^ transients generated by histamine stimulation in the presence of 1 mM extracellular Ca^2+^ shown in Fig. 2A, B are shaped by the Ca^2+^ influx from the extracellular medium (the store-operated Ca^2+^ entry (SOCE)) and the Ca^2+^ released from the intracellular stores such as ER and Golgi. Thus, to uncover potential selective modulation of Ca^2+^ signaling routes, and due to its importance in fine tuning cytosolic and mitochondrial targets, we focused our attention on the Ca^2+^ specifically released by the ER. Even small differences in this parameter, in fact, could profoundly impact not only on cytosolic but also on mitochondria-related activities, such as oxidative phosphorylation, through the contact sites between ER and mitochondria. To this aim, Ca^2+^ release from the intracellular stores was elicited by histamine in the absence of extracellular Ca^2+^ (in Krebs Ringer Buffer (KRB) supplemented with 2 mM EGTA and 20 μM CPA) to eliminate the contribution of the SOCE, and cytosolic Ca^2+^ transients were monitored with cytAEQ. To our surprise, the expression of the M protein either alone or along with the E protein (E + M) significantly and selectively reduced the Ca^2+^ released from the ER, while the expression of the E protein alone did not (peak values μM ± SEM: 3.176 ± 0.071, *n* = 78 for CTR; 2.918 ± 0.066, *n* = 78 for SARS-CoV-2 E; 2.475 ± 0.042, *n* = 78 for SARS-CoV-2 M; 2.508 ± 0.043, *n* = 78 for SARS-CoV-2 E + M), suggesting a selective modulation of the ER Ca^2+^ release by the M protein upstream of the E protein. Mitochondrial Ca^2+^ uptake was also measured in cells transfected with the E and the M protein, either alone or in combination (E + M), along with a vector encoding the low affinity mitochondrial photoprotein aequorin (mtAEQmut). Then, cells were stimulated with histamine (100 μM) to induce Ca^2+^ release from the ER. As shown in Fig. 2G, H, the mitochondrial Ca^2+^ peaks were significantly decreased in cells overexpressing the E and the M protein alone, while no differences were observed in cells co-expressing the E and M proteins (E + M) (peak values ± SEM: 65.54 ± 2.39, *n* = 67 for CTR; 54.11 ± 2.29, *n* = 67 for SARS-CoV-2 E; 48.77 ± 2.16, *n* = 67 for SARS-CoV-2 M; 59.83 ± 1.96, *n* = 67 for SARS-CoV-2 E + M). These data suggest an alteration of the Ca^2+^ handling systems by the SARS-CoV-2 proteins E and M, and a possible organelle specific effect when they are co-expressed together indicative of a reciprocal influence. As anticipated above, specific microdomains of high [Ca^2+^] at the interface between ER and mitochondria allows the latter to efficiently take up Ca^2+^ to sustain oxidative phosphorylation and ATP production, through the activation of the Ca^2+^-dependent dehydrogenases of the Krebs cycle [44]. Lowered mitochondrial Ca^2+^ uptake might depend on reduced release from the ER (as for the M protein) or on a reduced number of Ca^2+^ hotspots at the interface between ER and mitochondria. The data shown in Fig. 2E, F indicated that the first possibility might be true for the M protein but not for the E protein, suggesting that overexpression of the E protein lowered the mitochondrial Ca^2+^ peak through selective modulation of a different route. To further explore this possibility, we analyzed ER-mitochondria contact sites with SPLICS reporters [45–47]. As shown in Fig. 2I, L, where the SPLICS *puncta* were quantified on 3D rendered confocal stacks, the number of ER-mitochondria contact sites was significantly decreased in HeLa cells overexpressing the E protein, while no changes were measured upon overexpression of the M protein (values ± SEM: 84.99 ± 7.30, *n* = 35 for CTR; 61.80 ± 6.52, *n* = 32 for SARS-CoV-2 E; 98.62 ± 7.96, *n* = 33 for SARS-CoV-2 M; 65.88 ± 5.04, *n* = 26 for SARS-CoV-2 E + M). These results suggest the possibility that the reduced mitochondria Ca^2+^ uptake observed in Fig. 2G, H could be due to a reduction in the number of ER-mitochondria contact sites in the case of the E protein, while in the case of the M protein to the reduced release of Ca^2+^ from the ER. Co-expression of the E and the M protein (E + M) also led to interesting results: the M protein can work upstream or downstream the E protein based on whether the ER Ca^2+^ release or the ER-mitochondria contact sites are considered, respectively. Nevertheless, mitochondrial Ca^2+^ transients remained unchanged suggesting that modulation of additional mitochondria-specific factors might occur [48–52].

### Generation and purification SARS-CoV-2 E protein specific nanobodies

**Fig. 3:**
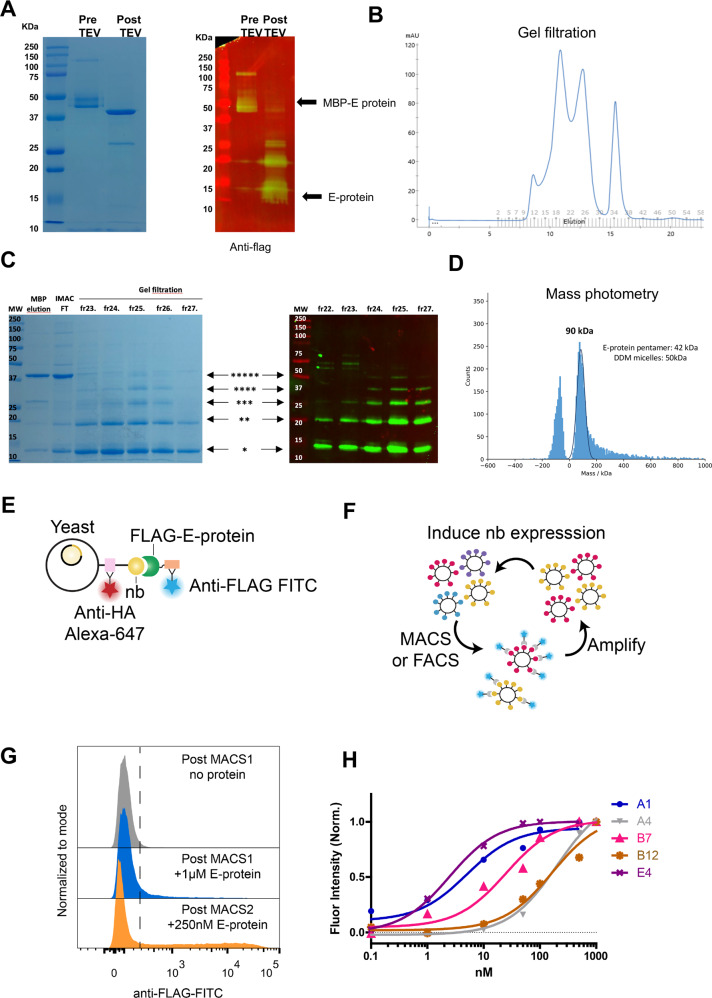
Expression of SARS-CoV-2 E protein fusion protein and nanobody isolation. **A** Coomassie blue-stained SDS-PAGE gel showing migration of molecular weight standards with masses in kDa indicated; elution from the Anylose column and after on-column cleavage with TEV. **B** Gel filtration profile of SARS-CoV2 E-protein on S200 10/300 size exclusion chromatography column, after TEV cleavage. **C** Coomassie blue-stained SDS-PAGE gel showing migration of molecular weight standards with masses in kDa indicated; elution from the MBP column, after cleavage with TEV protease; flow through after passing over Ni2+ IMAC column to remove His-tagged TEV; and individual gel filtration fractions across the peak corresponding to E-protein. E protein displays multiple oligomeric states on SDS-PAGE ranging from monomer to pentamer. In the right panel immunoblot with anti-FLAG antibodies confirming the identity of the bands as corresponding to FLAG-tagged E-protein. **D** Mass photometer analysis of TEV cleaved E protein shows that the molecular weight of the sample is compatible with a pentamer plus DDM micelles. **E**, **F** Nanobody isolation with purified E protein using yeast display and enrichment with magnetic-activated cell sorting (MACS). **G** Final round of single cell sorting using FACS and E protein. **H** Binding titration curves with E protein for the amplified single clones. Clones A1 and E4 were selected for further analysis.

Considering the potential role of the E protein viroporin as an antiviral and vaccine target, and to overcome its poor immunogenic potential possibly due to its small size and the lack of ectodomains for immune cell recognition [39, 40], we have generated SARS-CoV-2 E protein specific nanobodies and tested their efficacy in vitro by electrophysiological recordings and in living cells using mitochondrial Ca^2+^ transients. To this end, we have expressed SARS-CoV-2 E protein as a highly purified cleavable maltose binding protein (MBP) fusion (MBP-TEV-FLAG-E-protein). The fusion protein was expressed in *E. coli* (Rosetta DE3) and extracted from the membrane after solubilization with dodecyl maltoside (DDM). Gel electrophoresis showed a prominent band corresponding to the MBP-E protein fusion; cleavage with TEV and further purification by size exclusion chromatography yielded a monodisperse peak (Fig. 3A, B). After cleavage, the protein runs as a mix of different oligomerization states ranging from monomeric to pentameric on SDS- PAGE (Fig. 3C) consistent with previous reports for SARS-CoV-1 E-protein [26]. Mass photometry shows that the molecular weight of the purified protein is 90 kDa, which is consistent with an E protein pentamer embedded in a DDM micelle (Fig. 3D). Immunoblotting with an anti-FLAG antibody confirmed the identity of these bands as pure FLAG-tagged E-protein (Fig. 3C). We subsequently used the purifed E protein as bait to isolate nanobodies using yeast display from a library of yeast that express 1×10^8^ unique, synthetic nanobodies on their extracellular surface [53] (Fig. 3E, F). Three total rounds of enrichment were performed: an intial round of magnetic-activated cell sorting (MACS) using 1 µM E-protein as bait, a second round of MACS using 250 nM E-protein, and a final round of single cells sorting using FACS and 1 µM E-protein (Fig. 3G). Single clones were amplified and binding titrations with E-protein were performed [54] (Fig. 3H) and clones A1 and E4 were selected for further analysis.

### Effect of SARS-CoV-2 E protein nanobodies in vitro and in vivo

**Fig. 4:**
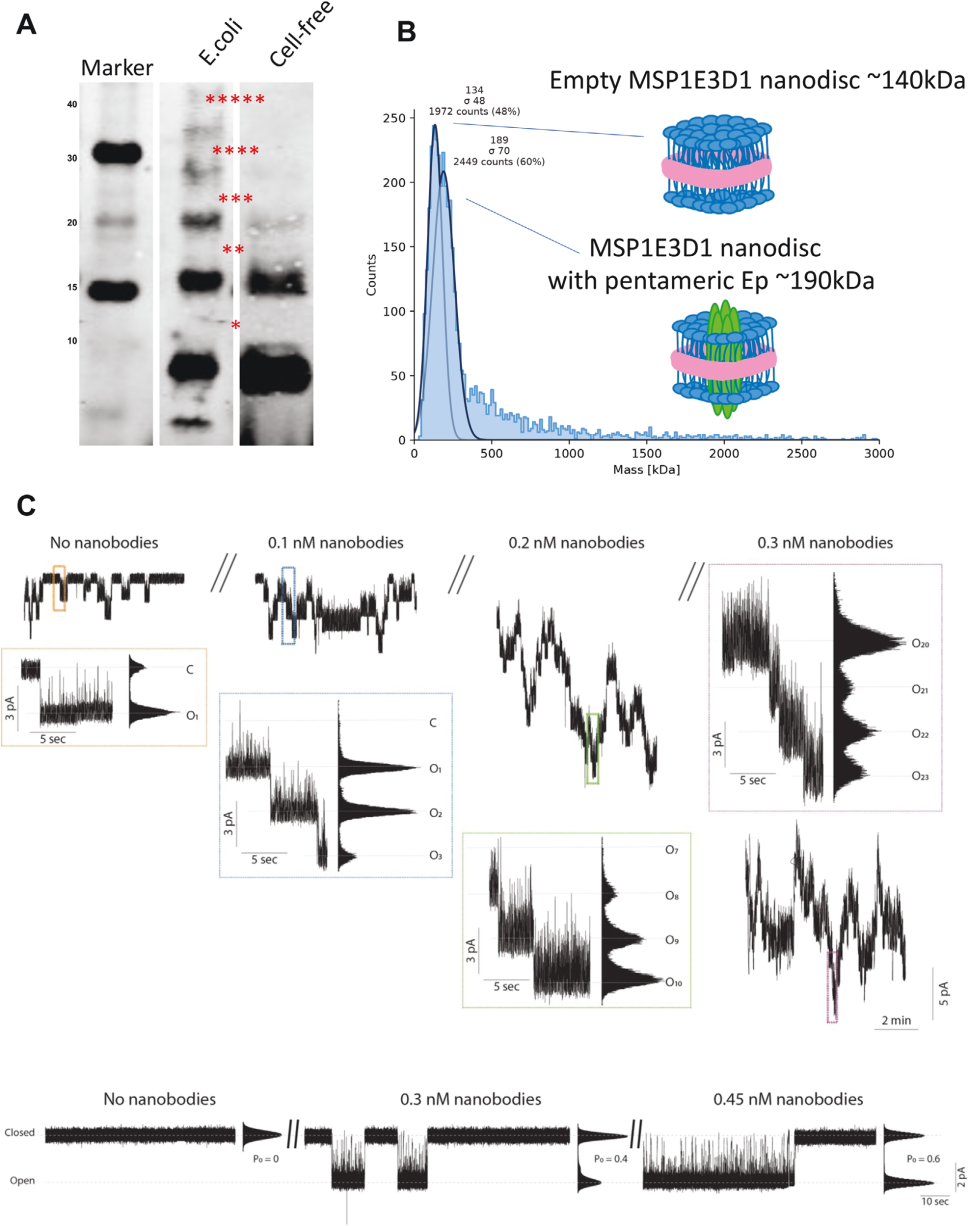
Effects of nanobody binding to soluble regions of SARS-CoV-2 E-protein. **A**, **B** Pentameric E-protein for ion channel recordings is produced using cell-free expression in the presence of lipid nano-discs and mass photometry showing pentameric SARS-CoV-2 E protein embedded in the lipid nano-disc membrane. Samples with cell-free protein production without SARS-CoV-2 E protein (no-vector) were tested on suspended membranes as a control for pore- or channel-forming contaminations. **C** Upper panel, multi-channel recordings at 0, 0.1, 0.2 and 0.3 nM nanobodies. Lower panel, single-channel recordings of E protein at nanobody concentration of 0.3 and 0.45 nM. Recordings were done in symmetrical buffer conditions using 250 mM KCl, 1 mM EGTA, and 10 mM HEPES, pH 7.4/KOH. Data were low-pass filtered with a 10 kHz Bessel filter and sampled at 100 kHz. After digitization, data were additionally filtered to achieve satisfactory signal-to-noise ratio using a digital Bessel filter at 0.1 kHz.

E protein for ion channel recordings was produced by cell-free expression of the E in the presence of lipid nanodiscs, resulting in pentameric SARS-CoV-2 E protein embedded in the lipid nanodisc membrane (Fig. 4A, B). Samples of cell-free protein production reactions without the SARS-CoV-2 E protein DNA (no-vector) were tested on suspended membranes as a negative control for pore- or channel-forming contaminants. As demonstrated by us and others for other small ion channels in lipid nanodiscs, SARS-CoV-2 E protein spontaneously transfers from the lipid nanodisc into the suspended lipid bilayer [55]. Recordings were performed in symmetrical buffer conditions of 250 mM KCl, 20 mM HEPES, pH 7.4 at constant membrane potential of −100 mV. Current traces of SARS-CoV-2 E protein show long closed dwell times (up to minutes) and intermediate long open states (seconds) with a single channel current amplitude of 3 pA (Fig. 4C). To avoid potential confusion between channel activation and the incorporation of additional channels the upper chamber (trans-chamber, connected to ground) was washed extensively to remove excessive SARS-CoV-2 E protein as soon as initial channel activity was monitored. We performed ion channel recordings of SARS-CoV-2 E protein in suspended membranes starting in the absence of nanobodies and increasing the nanobody concentration within the experiment in three steps from 0.1 to 0.2 and finally to 0.3 nM. The time series in Fig. 4 (upper part) shows a concentration-depended activation of SARS-CoV-2 E protein generated currents. At the 0.3-nM concentration step, we extensively washed the sample chamber to make sure the effects were not based on non-specific interactions between the nanobody and the membrane or the SARS-CoV-2 E protein. Before and after washing we were able to record an average of 15–17 active channels. To gain more detailed understanding on the effect of the nanobody binding on the open probability of SARS-CoV-2 E protein we sought to perform single-channel recordings. The data in Fig. 4C (upper panel) show that the open probability is increased from the closed state (P_0_ = 0) to P_O_ = 0.4 at 0.3 nM and to P_O_ = 0.6 at a nanobody concentration of 0.45 nM (Fig. 4C, lower panel). These data clearly indicate that interaction with specific nanobodies can modulate SARS-CoV-2 E protein activity.

**Fig. 5:**
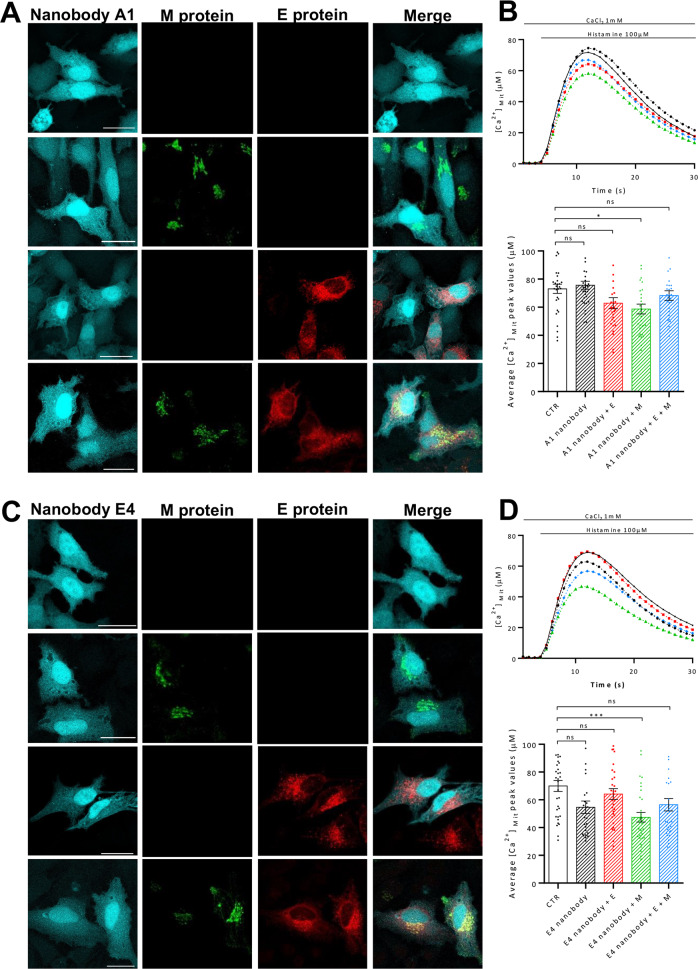
The A1 and E4 nanobodies reverse the effect of the Sars-CoV-2 E protein on the host cell’s calcium handling. Representative images of HeLa cells expressing both the A1 (**A**)/E4 (**C**) nanobody and the viral proteins/empty vector. The E and M proteins were detected with a rabbit polyclonal antibody anti-FLAG tag and a mouse monoclonal antibody anti-His tag, respectively. The presence of the nanobody was revealed by the CFP. Images were acquired with a Leica SP5 confocal microscope with a HCX PL APO 100.0×1.40 OIL objective upon illumination with laser at the wavelength of 405 nm, 488 nm, and 594 nm. Scale bar = 25 μM. To assess the effect of the A1 (**B**) and E4 (**D**) nanobody, HeLa cells were transfected with the E and M proteins, either alone or in co-transfection, the mitochondrial-targeted mutated aequorin, and the nanobody of interest. Then, cells were stimulated with 100 μM histamine to induce the mitochondrial Ca^2+^ uptake. From each calcium trace (in the upper panel the average of at least 25 independent measurements obtained from three independent transfections, mean ± SEM), the peak was calculated and plotted in the lower panel (mean ± SEM). One-way ANOVA = ****p < 0.0001. Tukey’s multiple comparisons test was applied for multiple comparisons (*p < 0.05; ***p < 0.001 versus CTR).

The results presented above demonstrated the ability of our purified nanobodies to modulate the ion channel activity of the purified SARS-CoV-2 E protein. Therefore, we decided to test these nanobodies in living cells, by assessing whether the expression of selected nanobodies could reverse the reduction of mitochondria Ca^2+^ uptake observed upon the overexpression of the E protein. To this end, mitochondrial Ca^2+^ uptake was measured in HeLa cells expressing the E and the M protein, either alone or in combination (E + M), along with a vector encoding for mtAEQmut and the selected nanobodies, namely A1 and E4. Mitochondrial Ca^2+^ transients were recorded upon stimulation with histamine (100 μM) to induce Ca^2+^ release from the ER, and the results are showed in Fig. 5. The expression of the A1 and the E4 nanobodies, along with the E, the M or the E + M SARS-CoV-2 proteins, was verified by immunofluorescence analysis (Fig. 5A, E, respectively). In Fig. 5B and D, the mitochondrial Ca^2+^ transients (top panel) and the quantification of the mitochondrial Ca^2+^ peaks (lower panel) are shown. The obtained results show that the Ca^2+^ uptake was not significantly altered in cells overexpressing the A1 (peak values μM ± SEM; 73.06 ± 3.3.3, *n* = 30 for CTR; 75.60 ± 2.839 *n* = 30 for the cells expressing the A1 nanobody alone; 62.85 ± 3.901, *n* = 29 for A1 + SARS-CoV-2 E; 56.83 ± 3.099, *n* = 29 for A1 + SARS-CoV-2 M; 68.27 ± 3.417, *n* = 3.417, *n* = 30 for A1 + SARS-CoV-2 E + M) and the E4 nanobodies (peak values μM ± SEM: 71.67 ± 4.171, *n* = 34 for CTR; 65.87 ± 6.628, *n* = 31 for the cells expressing the E4 nanobody alone; 64.15 ± 3.859, *n* = 34 for E4 + SARS-CoV-2 E; 47.41 ± 3.459, *n* = 33 for E4 + SARS-CoV-2 M; 56.42 ± 4.4478, *n* = 32 for E4 + SARS-CoV-2 E + M) alone. Interestingly, the presence of A1 as well as E4 specific nanobody was able to fully rescue the decrease in mitochondrial Ca^2+^ uptake induced by the SARS-CoV-2 E protein, without affecting the mitochondrial Ca^2+^ uptake induced by the overexpression of the M and the E + M proteins, confirming that targeting the E protein could represent an important therapeutic approach for SARS-CoV-2 infection.

## Discussion

In the present study we focused on two “neglected” SARS-CoV-2 structural proteins, namely the E and M. They are present on the viral envelope and form the structural components of virions along with the S and the N protein and are fundamental for the assembly of viruses through homotypic or heterotypic interactions [56, 57]. Interactions between the E and M proteins occur in the ERGIC and are important for viral assembly [58, 59], while the homo-oligomerization of the E proteins leads to the formation of ion conductive pores in the viral membrane and viral assembly and budding at sites of intracellular transport, i.e., the ER, the Golgi complex and ERGIC [57]. We have explored their ability to subvert cellular Ca^2+^ handling upon overexpression in mammalian HeLa cells and found that the M and E protein are mainly localized in the ER, in secretory compartments, and the Golgi, impinging on mitochondria Ca^2+^ transients by selectively affecting the release of Ca^2+^ from the ER and the contact sites between the ER and mitochondria, respectively. If and how they can influence each other in modulating the cellular Ca^2+^ signaling differently, is still unclear. As far as the ER Ca^2+^ release is concerned, the M protein appears to play a major role, while the E protein appears to selectively affect ER-mitochondria tethering. No major changes in mitochondrial Ca^2+^ uptake are observed upon co-expression of the two proteins, suggesting the possibility that additional mitochondria-related functions might be modulated. We further focused our attention on the small E protein suggested to be involved in releasing Ca^2+^ out of the ER [56] and generated and isolated selective nanobodies to target its function. Electrophysiological recordings clearly showed the ability of the selected nanobodies to modulate the ion conductance of the E protein. The presence of two nanobodies i.e., clone A1 and E4, in mammalian cells along with the E protein was sufficient to efficiently revert the phenotype observed on mitochondrial Ca^2+^ uptake, suggesting that the E protein might be an ideal target for vaccine and drug development but also a target for immunotherapies not only triggering a directed immune response but also directly interfering with the regulation and hence function of the target protein to fight SARS-CoV-2 infection and COVID-19 disease.

## Methods

### Protein purification

*Escherichia coli* Rosetta (DE3) strain was grown at 20 °C in 2YT media after induction with 0.3 mM IPTG. Bacteria were collected and resuspended in 20 mM HEPES (pH 7.5), 150 mM NaCl and a tablet of complete (EDTA-free) protease inhibitor (Roche). Cells were disrupted by sonication, followed by a 20-min centrifugation at 18,000 ×g. The membrane pellet was solubilized in 20 mM HEPES (pH 7.5), 150 mM NaCl, 20 mM imidazole, 1%(w/v) DDM for 1 h at 4 °C. Insoluble material was removed by centrifugation at 100,000 ×g for 30 min. The solubilized fraction was loaded onto an Amylose resin (New England Biolabs) preequilibrated with 20 mM HEPES (pH 7.5), 150 mM NaCl and 0.02% DDM. Proteins were eluted with 10 mM maltose in the same buffer. Maltose binding protein was cleaved using TEV protease ON (1:25).

The cleaved protein was further purified from free MBP by loading on a Superdex 200 Increase 10/300 GL size-exclusion column (GE Healthcare Life Sciences) in gel filtration buffer (20 mM HEPES pH 7.5, 150 mM NaCl, DDM 0.02%). Purified E protein was incorporated into lipid nanodiscs with a 1:300:5 molar ratio of protein: POPC: membrane scaffold protein 1D1 (MSP1D1). This mixture was incubated at 4 °C for 2 h with gentle agitation. Reconstitution was initiated by removing detergent by incubating with Bio-beads (Bio-Rad) at 4 °C overnight with constant rotation. Bio-beads were removed and the nanodisc reconstitution mixture was bound again to Anti-FLAG resin (Biolegend) preequilibrated with 20 mM HEPES (pH 7.5), 150 mM NaCl at 4 °C for 2 h to remove free nanodiscs. The protein was eluted with 100 μg/mL FLAG peptide. The eluted protein was further purified by loading on a Superdex 200 Increase 10/300 GL size-exclusion column (GE Healthcare Life Sciences) in gel filtration buffer (20 mM HEPES pH 7.0 and 150 mM NaCl).

### Mass Photometry (MP, iSCAMS)

Mass spectrometry experiments were performed with a Refeyn OneMP (Refeyn Ltd.). Data acquisition was performed using AcquireMP (Refeyn Ltd. 172 v2.3). Samples were evaluated with microscope coverslips (70 × 26 174 mm). The coverslips were washed with ddH_2_O and isopropanol. A silicone template was placed on top of the coverslip to form reaction chambers immediately prior to measurement. The instrument was calibrated using NativeMark Protein Standard (Thermo Fisher). 10 μL of fresh room temperature buffer was pipetted into a well, and the focal position was identified and locked. For each acquisition 1 μL of the protein (at a concentration of 200 nM) was added to the well and thoroughly mixed. MP signals were recorded for 60 s to allow recording of at least 2 × 10^3^ individual protein binding events. The data were analyzed using the DiscoverMP software.

### Nanobody Purification

Production and purification of the nanobodies was carried out as described by Pardon and colleagues [60]. Briefly, nanobody overexpression was carried out in *E. coli* pLys transformed at 37 °C in Terrific Broth supplemented with 100 μg/mL ampicillin, 0.1% glucose and 1 mM MgCl_2_, until OD_600_ reached 0.7. Nanobody expression was then induced by adding 1 mM IPTG, and the culture was grown overnight at 28 °C. Following bacterial cell harvesting by centrifugation, the pellet was resuspended in ice-cold TES buffer (0.2 M Tris pH 8.0, 0.5 mM EDTA and 0.5 M sucrose) and incubated for 1 h on ice with shaking. Periplasmic extract was then obtained by adding two volumes of TES/4 buffer (25% v/v TES in ddH_2_O) and incubating the suspension on ice for 45 min with shaking. His-tagged nanobodies were purified from soluble periplasmic extract by IMAC (HisTrap HP 1 mL column, GE Healthcare), washing extensively the column with 50 mM sodium phosphate pH 7.0, 1 M NaCl, and then eluting the desired proteins with 50 mM sodium phosphate pH 7.0, 150 mM NaCl and 0.5 M imidazole. The most pure fractions containing nanobodies, as assessed by SDS-PAGE, were pooled together and buffer exchanged into the final storage buffer (30 mM HEPES pH 7.5, 150 mM NaCl, 10% glycerol).

### Cell cultures and transfection

HeLa cells were grown in Dulbecco’s modified Eagle’s medium (DMEM) high glucose, 110 mg/L sodium pyruvate (Gibco; Cat.# 41966-029), supplemented with 10% (vol/vol) Fetal Bovine Serum (FBS) (Gibco; Cat.# 10270-106) and 100 μg/ml Penicillin–Streptomycin (EuroClone; Cat.# ECB3001D), at 37 °C in a 5% CO2 atmosphere. Twenty hours before transfection, cells were seeded onto 13-mm glass coverslips (30 000 cells/well) (for immunofluorescence analyses) or into a 6-multiwell plate (350 000/well) (for western blot and Ca^2+^ analyses). Transfection was carried out with the Ca^2+^ phosphate procedure, using 4 µg of total DNA for each 13-mm glass coverslip and 12 µg for each well of the 6-multiwell plate. For Ca^2+^ measurement cells were co-transfected with aequorin constructs targeted to different cell compartments (cytAEQ, erAEQ, mtAeqmut) and pcDNA3 empty vector or plasmids encoding the protein E or M. The growth medium was replaced with fresh medium right before transfection. After eight hours, cells were washed at least three times with Dulbecco’s Phosphate Buffered Saline (D-PBS) (EuroClone; Cat.# ECB4004L) and fresh medium was added.

### Western blotting analysis

Forty-eight hours after transfection, proteins from Hela cells plated into 6-multiwell plate were extracted by solubilizing cells in ice-cold RIPA lysis buffer (150 mM NaCl, 50 mM Tris–HCl, pH 7.4, 1 mM ethylene glycol tetra acetic acid/Tris (EDTA) pH 7.4, and 1% Triton X-100, 1 mM phenylmethylsulfonyl fluoride (PMSF) and a cocktail of protease inhibitors (Sigma; Cat.# 8340)). Cell lysates were collected after 20 min of centrifugation at 15000 g at 4 °C. The total protein amount was quantified by the Bradford assay by the Quick Start™ Bradford Protein Assay (Biorad; Cat.# 500-0201) following the manufacturer’s instruction. 15 μg of protein for each sample were loaded on a 15% SDS-PAGE Tris–HCl gel, transferred onto polyvinylidene fluoride (PVDF) membrane (Biorad; Cat.# 162-0177). The membrane was blocked for at least 1 h at room temperature, using 5% non-fat dried milk (Euroclone; Cat.# ERM180500) in a Tris-buffered saline solution with 0.1% Tween® 20 detergent (TBS-T) (20 mM Tris-HCl, pH 7.4, 150 mM NaCl) and incubated overnight with the specific primary antibody at 4 °C. Dilutions in TBS-T of the specific primary antibodies were used: anti-FLAG tag raised in rabbit (1:1000, Proteintech, Cat.# 20543-1-AP), anti-His tag raised in mouse (1:5000, Proteintech, Cat.# 66005-1-Ig), and anti-tubulin antibody raised in mouse (1:30000, Sigma-Aldrich; Cat.# A5441). The bands detection was carried out by incubation with secondary horseradish peroxidase-conjugated antibodies (Santa Cruz Biotech: goat anti-Mouse IgG-HRP, Cat.# sc-2005; and Mouse anti-Rabbit IgG-HRP; Cat.# sc-2357; 1:4000 in TBS-T) for 2 h at RT, followed by incubation with the chemiluminescent reagent Luminata Classico HRP substrate (Merck Millipore; Cat.# WBLUO500).

### Immunocytochemistry analysis

Forty-eight hours after transfection, HeLa cells were processed for immunofluorescence. Cells were fixed with a 3.7% formaldehyde (formaldehyde stock solution 37% in H_2_O) (Sigma-Aldrich; Cat.# F8775) solution in D-PBS for 20 min and washed three times with D-PBS. Cell permeabilization was performed by a 10 min-long incubation in a 0.1% Triton X-100 (PanReac AppliChem; Cat.# A1388) solution in D-PBS, followed by three washes (each 10 min long) in a 1% gelatine (Type B from bovine skin) (Sigma-Aldrich; Cat.# G9382) solution in D-PBS at room temperature for 15 min. The coverslips were then incubated for 2 h at room temperature with the specific primary antibody diluted in D-PBS: anti-FLAG tag raised in rabbit (1:50, Proteintech; Cat.# 20543-1-AP); anti-His tag raised in mouse (1:50, Proteintech; Cat.# 66005-1-Ig); anti-KDEL raised in rabbit (1:100, abcam; Cat.# ab2898); anti-TOM20 raised in rabbit (1:100, Santa Cruz Biotech; Cat.# sc-11415); anti-TGN-46 raised in rabbit (1:100, abcam; Cat.# ab16059). Further washing steps with gelatin were repeated to remove the excess of primary antibody. The staining was revealed by the incubation with specific Alexa Fluor secondary antibodies with a dilution 1:100 in D-PBS (Thermo Fisher: goat anti-rabbit IgG Alexa Fluor 405, Cat.# A-913925; goat anti-rabbit IgG Alexa Fluor 594, Cat.#A11036; goat anti-mouse IgG Alexa Fluor 488, Cat.#A1101) for 45 min at room temperature. Coverslips were mounted using Mowiol 40-88 (Sigma-Aldrich; 81386), after a final wash with D-PBS. Fluorescence was detected with a Leica SP5 confocal microscope and analysed by ImageJ software.

### Aequorin Ca^2+^ measurements

Cytosolic, mitochondrial and ER Ca^2+^ measurements were carried out on a PerkinElmer EnVision plate reader equipped with a two-injector unit, as previously reported [61, 62].

### Cell free protein expression, purification and Western-blot analysis

Cell free expression of Ep-CoV-2 was done using the MembraneMax Protein Expression System (ThermoFisher Scientific, Waltham, MA, USA) following manufacturer’s instructions. In brief, 1 µg of template DNA (pET24Δlac/Ep-CoV-2) was added to the transcription/translation reaction mixture in the presence of pre-assembled MSP1E3D1-His DMPC nanodiscs (Cube Biotech GmbH, Monheim, Germany). After initial incubation for 30 min at 37 °C and 1250 rpm, feeding buffer was added and incubation was continued for additional 1.5 h.

The reaction mixture was then added to a 0.2 mL HisPur Ni-NTA Spin column (ThermoFisher Scientific, Waltham, MA, USA), pre-equilibrated in PBS (phosphate buffered saline), adjusted to pH 7.4, with 10 mM imidazole, and incubated at constant shaking for 30 min at 37 °C. Flow through was collected at 700 × g for 2 min, the column was then washed three times with 400 µL PBS adjusted to pH 7.4 with 25 mM imidazole, and finally proteins were eluted three times using 200 µL PBS adjusted to pH 7.4, with 250 mM imidazole. For western-blot analysis protein concentration was increased aprox. 30-fold using an Amicon Ultra-0.5 mL 100 kDa cut-off (Merck KgaA, Darmstadt, Germany) to a residual volume of 20 µL.

For western blotting, proteins were diluted in 4x LDS sample buffer (ThermoFisher Scientific) and separated using a Novex 4–12% Bis-Tris LDS Gel (ThermoFisher Scientific) in MES running buffer (ThermoFisher Scientific) at constant 150 V until the blue loading dye front reached the end of the gel. Proteins were transferred onto a Nitrocellulose membrane (ThermoFisher Scientific) using the iBlot2 dry blotting system (ThermoFisher Scientific). Membranes were blocked for 1 h at RT using Pierce™ Clear Milk Blocking Buffer (ThermoFisher Scientific). Blots were incubated overnight at 4^o^C with mouse anti-FLAG tag mAB (Cell Signaling Technology, Danvers, MA, USA) at a final dilution of 1:1000 in blocking buffer. Blots were washed three times in Tris-buffered saline solution with 0.1% Tween for 5 min, incubated with Goat-anti mouse IRDye 680 mAB diluted 1:15,000 in blocking buffer for at least 1 h at RT or overnight at 4 °C and imaged using a LiCor Odyssey imaging system (LI-COR, Lincoln, NE, USA).

### Ion channel recordings

Recordings of Ep-CoV-2 mediated channel activity were done in horizontal suspended lipid bilayers by voltage-clamp recordings. DPhPC (1,2-diphytanoyl-sn-glycero-3-phosphocholine) and DPhPS (1,2-diphytanoyl-sn-glycero-3-phospho-L-serine) were obtained from Avanti Polar Lipids (Alabaster, AL, USA) in chloroform solution. DPhPC and DPhPS were mixed 50/50 and 100 µL of the final solution was dried under a constant nitrogen stream and re-suspended in 100 µL n-decane to reach a final concentration of 25 mg/ml. Lipid bilayers were formed on the 50 µm cavity of a MECA4 chip (Ionera Technologies GmbH, Freiburg, Germany) using the pseudo-painting bubble technique resulting in stable lipid bilayer with a typical membrane capacitance of 40–60 pF. Nanodiscs loaded with Ep-CoV-2 were added to the *trans*-side, i.e., the upper chamber with the reference Ag/AgCl electrode connected to ground on the HEKA EPC10-USB (HEKA Instruments) and voltage bias was applied at the Ag/AgCl electrode in the microcavity on the cis-side in reference to the ground electrode. After addition of 1-2 µL of the protein solution incorporation was typically monitored within 20–30 min. The *trans*-chamber was perfused with 10-20 sample chamber volumes of buffer to remove unbound proteins using a gravity driven perfusion system with vacuum suction. Recordings were done in symmetrical buffer conditions using 250 mM KCl, 1 mM EGTA, and 10 mM HEPES, pH 7.4/KOH. Data were low-pass filtered with a 10 kHz Bessel filter and sampled at 100 kHz. After digitization, data were additionally filtered to achieve satisfactory signal-to-noise ratio using a digital Bessel filter at 0.1 kHz.

### Statistical analysis

All the data are representative of at least three independent experiments unless otherwise indicated. Values are expressed as mean ± SEM. Statistical significance was determined using the multiparametric one-way ANOVA test. A p value ≤0.05 was considered statistically significant.

## Supplementary information

Reproducibility checklist

Supplemental Material Figures S1-3

Full Scan WB

## Data Availability

No datasets were generated or analyzed during the current study. Additional data are available from the corresponding author on reasonable request.
