# Supplementary material for: Perturbation of the host cell Ca2+ homeostasis and ER-mitochondria contact sites by the SARS-CoV-2 structural proteins E and M
Source: Cell Death Dis. 2023 Apr 29;14(4):297. doi: 10.1038/s41419-023-05817-w (PMC10148623; doi:10.1038/s41419-023-05817-w)
Supplement: Supplementary file 2 — Supplemental Material Figures S1-3 [file 41419_2023_5817_MOESM2_ESM.docx]

**Perturbation of the host cell Ca^2+^ homeostasis and ER-mitochondria contact sites by the SARS-CoV-2 structural proteins E and M**

**Supplementary Figures**

**Supplementary Figure 1**

**
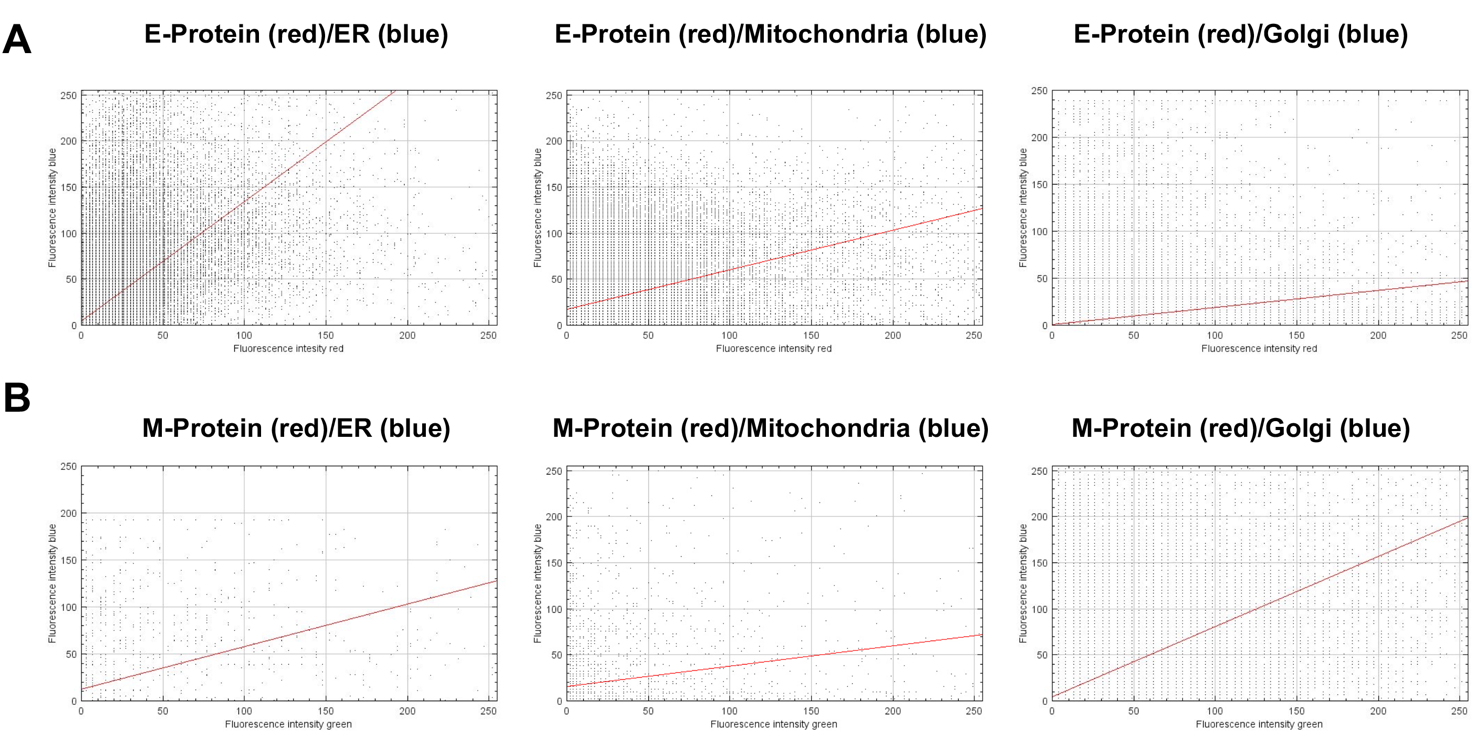
**

**Figure S1.** **Colocalization analyses of the SARS-Cov-2 structural proteins.** Colocalization analysis of confocal images of the E (A) and M (B) structural proteins with subcellular compartments were prformed using the ImageJ software (free software released by NIH, https://imagej.nih.gov/ij) and the JACOP plugin <https://imagej.nih.gov/ij/plugins/track/jacop2.html>), and representative cytofluorograms are reported.

**Supplementary Figure 2**

**
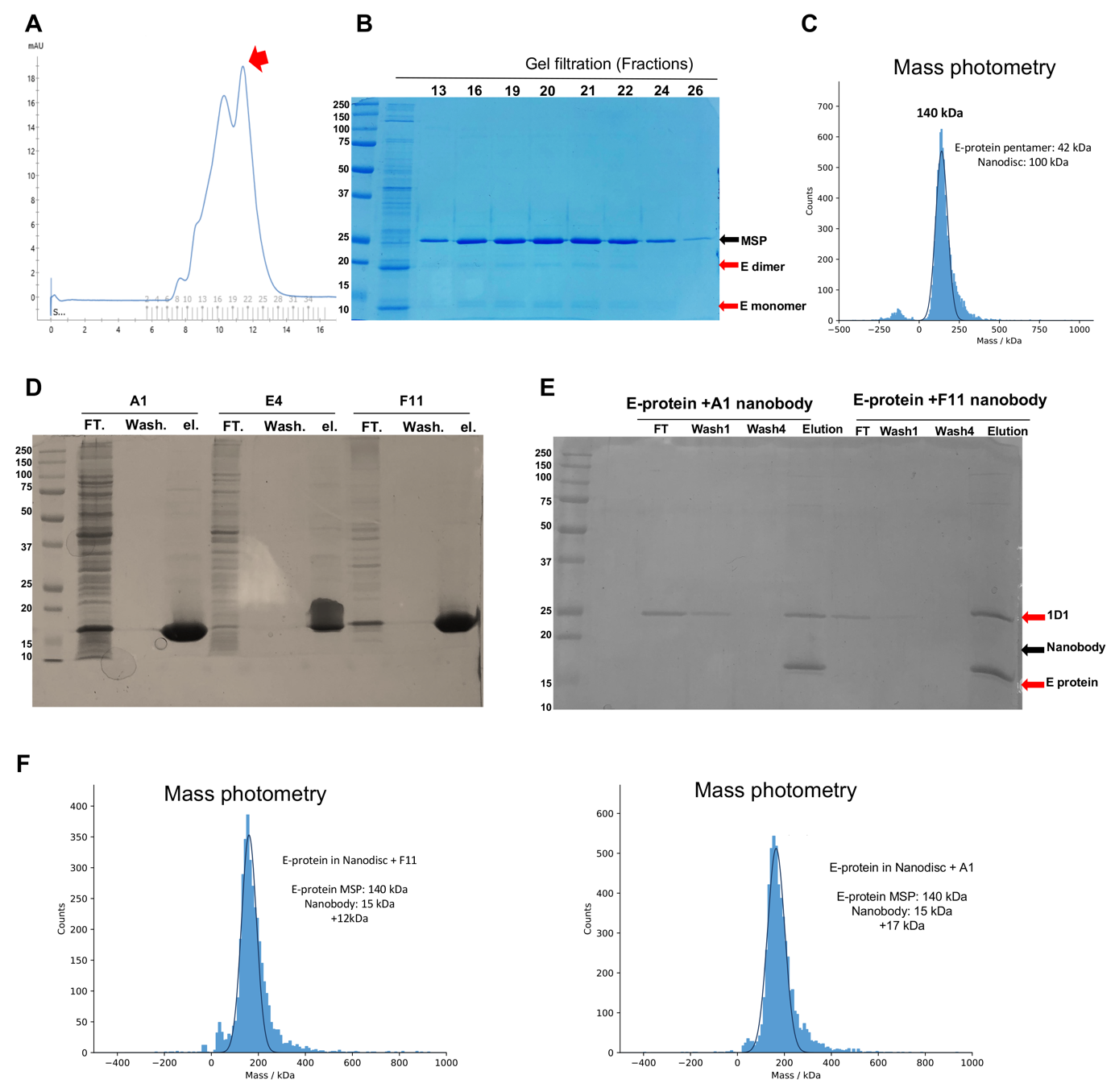
**

**Figure S2. Incorporation of SARS-CoV-2 E protein into 1D1 nanodisc and interaction with nanobodies.** (A) Gel filtration profile of SARS-CoV2 E-protein on S200 10/300 size exclusion chromatography column, after nanodisc incorporation. (B) Coomassie blue-stained SDS-PAGE gel showing migration of molecular weight standards with masses in kDa indicated; gel filtration fractions showing the presence of 1D1 MSP, and E protein (C) Mass photometer analysis of E protein in nanodisc shows that the molecular weight of the sample is compatible with a pentamer plus 1D1 nanodisc. (D) Coomassie blue-stained SDS-PAGE gel showing nanobody purification. (E) Coomassie blue-stained SDS-PAGE gel showing migration of molecular weight standards with masses in kDa indicated; nickel purification of E protein MSP plus nanobody (F) Mass photometer analysis of E protein in nanodisc plus nanobody F11 and A1 shows that the molecular weight of the sample is compatible with a E protein pentamer in MSP nanodisc plus the nanobody.

**Supplementary Figure 3**


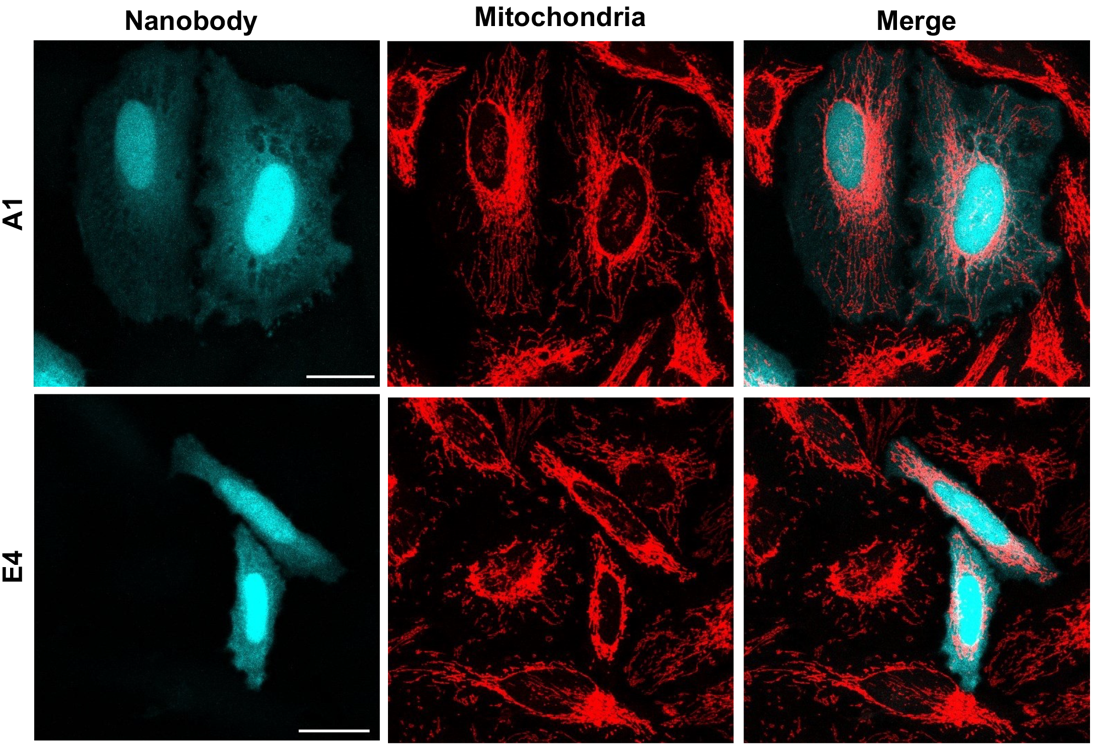


**Figure S3. Nanobody overexpression does not affect the mitochondrial morphology.** Representative confocal images of HeLa cells transfected with the indicated nanobodies, A1 and E4. CFP-tagged nanobodies are shown in cyan, while mitochondria were stained with Mitotracker red fluorescent dye (Thermo Fisher). Images were acquired with a Leica SP5 confocal microscope with a HCX PL APO 100.0x1.40 OIL objective upon illumination with laser at the wavelength of 405 nm and 560 nm. Scale bar = 25 μM.
