# Supplementary figures and images for: Perturbation of the host cell Ca2+ homeostasis and ER-mitochondria contact sites by the SARS-CoV-2 structural proteins E and M
Source: Cell Death Dis. 2023 Apr 29;14(4):297. doi: 10.1038/s41419-023-05817-w (PMC10148623; doi:10.1038/s41419-023-05817-w)

Full Blot Figure 1E

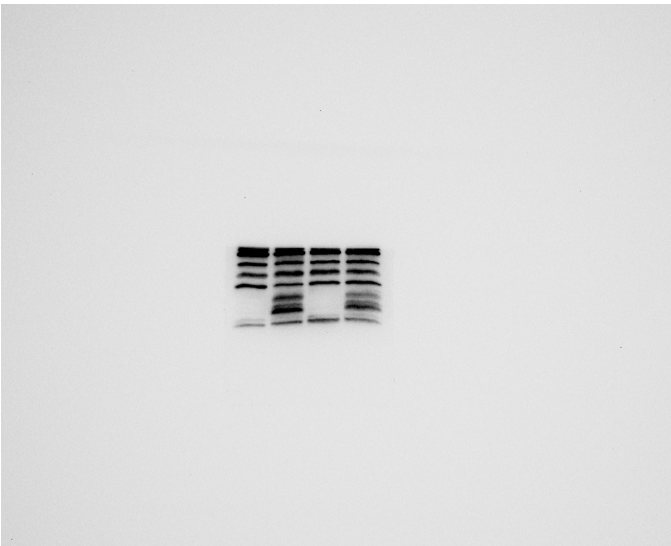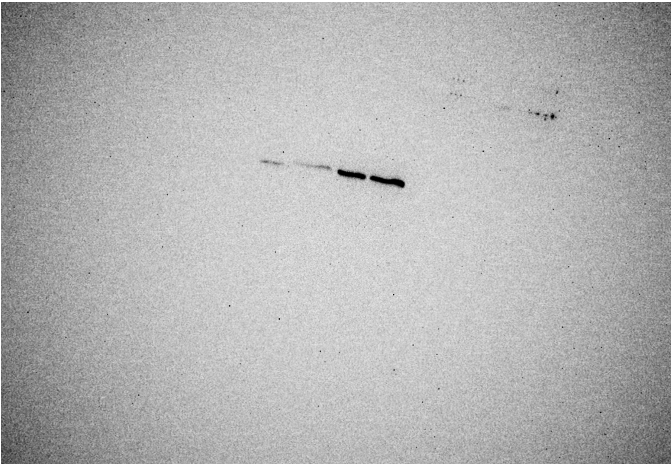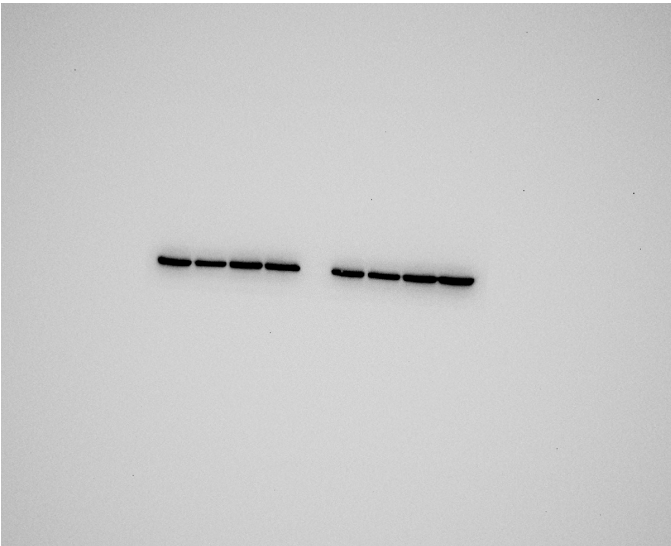

Full Blot Figure 4A

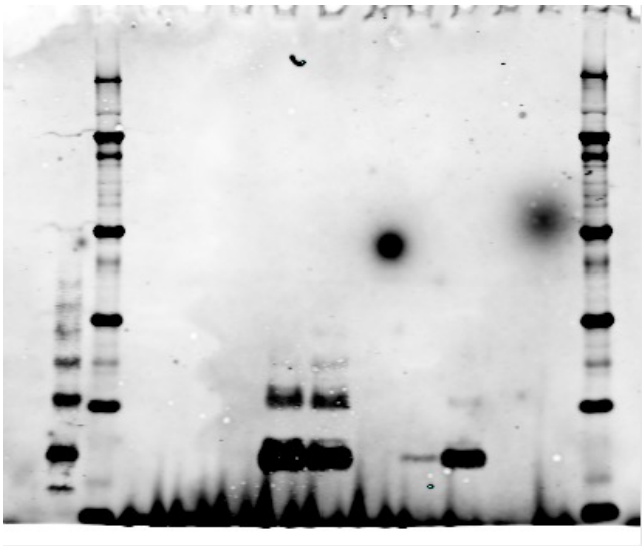

Supplement: Supplementary file 3 — Full Scan WB [file 41419_2023_5817_MOESM3_ESM.pdf]
